# Automated detection of heuristics and biases among pathologists in a computer-based system

**DOI:** 10.1007/s10459-012-9374-z

**Published:** 2012-05-23

**Authors:** Rebecca S. Crowley, Elizabeth Legowski, Olga Medvedeva, Kayse Reitmeyer, Eugene Tseytlin, Melissa Castine, Drazen Jukic, Claudia Mello-Thoms

**Affiliations:** 1 Department of Biomedical Informatics, University of Pittsburgh School of Medicine, UPMC Shadyside Cancer Pavilion—Room 313, 5150 Centre Avenue, Pittsburgh, PA 15232 USA; 2 Department of Pathology, University of Pittsburgh School of Medicine, Pittsburgh, PA USA; 3 James A. Haley VA Medical Center, Tampa, FL USA; 4 Department of Pathology and Cell Biology, University of South Florida, Tampa, FL USA; 5 Department of Dermatology, University of South Florida, Tampa, FL USA

**Keywords:** Biases, Clinical competence, Cognition, Diagnostic errors, Diagnostic reasoning, Medical education, Educational technology, Heuristics, Metacognition, Pathology

## Abstract

The purpose of this study is threefold: (1) to develop an automated, computer-based method to detect heuristics and biases as pathologists examine virtual slide cases, (2) to measure the frequency and distribution of heuristics and errors across three levels of training, and (3) to examine relationships of heuristics to biases, and biases to diagnostic errors. The authors conducted the study using a computer-based system to view and diagnose virtual slide cases. The software recorded participant responses throughout the diagnostic process, and automatically classified participant actions based on definitions of eight common heuristics and/or biases. The authors measured frequency of heuristic use and bias across three levels of training. Biases studied were detected at varying frequencies, with availability and search satisficing observed most frequently. There were few significant differences by level of training. For representativeness and anchoring, the heuristic was used appropriately as often or more often than it was used in biased judgment. Approximately half of the diagnostic errors were associated with one or more biases. We conclude that heuristic use and biases were observed among physicians at all levels of training using the virtual slide system, although their frequencies varied. The system can be employed to detect heuristic use and to test methods for decreasing diagnostic errors resulting from cognitive biases.

## Introduction

Human judgments made under uncertainty have been shown to be subject to a wide array of cognitive heuristics (mental “short-cuts” or “rules of thumb”) that may produce systematic biases (Tversky and Kahneman 1974). Many authors have suggested that use of cognitive heuristics by physicians may contribute to medical errors (Bornstein and Emler 2001; Croskerry 2003; Dawson and Arkes 1987; Elstein 1999; Graber et al. 2002; Kempainen et al. 2003), supported by some empirical studies of heuristic use among medical students, residents, and attending physicians (Christensen-Szalanski and Bushyhead 1981; Kassirer and Kopelman 1989; Kern and Doherty 1982; Payne and Crowley 2008; Voytovich et al. 1985; Wallsten 1981). An alternative view is that heuristic use can be a highly effective strategy, producing “fast and frugal” judgments (Gigerenzer and Gaissmaier 2011) of potentially high accuracy in clinical contexts (Eva and Norman 2005). This dualistic view on the underlying nature of these shortcuts reflects the fact that they may lead to a correct diagnosis, in which case they are termed “heuristics”, or to an incorrect one, in which case they are termed “biases” or “heuristic biases”. Furthermore, heuristics like viability (Lago 2008) and representativeness, properly used, are associated with a fast, exemplar-based non-analytical model of thinking (Norman et al. 2007) which has been linked to expert reasoning.

Examples of observed biases include *anchoring with insufficient adjustment* (Croskerry 2002; Ellis et al. 1990; Richards and Wierzbicki 1990; Tversky and Kahneman 1974), *availability bias* (Dawson and Arkes 1987; Poses and Anthony 1991; Tversky and Kahnamen 1980), *base rate neglect* (Christensen-Szalanski and Bushyhead 1981), *representativeness* (Dawson and Arkes 1987; Payne and Crowley 2008; Tversky and Kahnamen 1980), *confirmation bias* (Croskerry 2002; Nickerson 1998; Pines 2006), and *satisficing (premature closure)* (Simon 1956; Redelmeier and Shafir 1995).

Recent work has focused on categorizing these heuristics and biases (Campbell et al. 2007; Croskerry 2002; Graber et al. 2002), identifying contexts in which they may apply, (Dawson and Arkes 1987; Elstein 1999) and providing examples relevant to clinical medicine (Bornstein and Emler 2001; Kempainen et al. 2003; Pines 2006). Many authors have suggested that reducing the use of these shortcuts may decrease biases and errors (Bornstein and Emler 2001; Croskerry 2003; Graber et al. 2002). However, the question of whether heuristic use can or should be altered is controversial (Eva and Norman 2005), as attempts to debias human judgments have produced mixed results (Fischhoff 1982; Fong et al. 1986; Hirt and Markman 1995; Hodgkinson et al. 1999; Koriat and Bjork 2006; Kosonen and Winne 1995; McKenzie 2006; Mumma and Wilson 1995; Wolf et al. 1988). It seems well accepted that heuristics lead to accurate responses in the majority of cases (Eva and Norman 2005; Gigerenzer and Gaissmaier 2011).

Many gaps remain in our understanding of heuristics and biases as they apply to clinical reasoning. For example, little is known about the frequency of any heuristic and/or bias in clinical reasoning, the relative frequency among heuristics and biases, the ratio of appropriate heuristic use to bias, the degree to which individuals may be susceptible to biases, and the relationship of heuristic use and biases to the level of medical training. Our incomplete understanding of heuristic use in clinical reasoning limits our ability to develop educational and patient safety interventions (Croskerry 2003; Graber et al. 2002).

In previous work, we have implemented a unique intelligent medical training system (Crowley and Gryzbicki 2006; Crowley and Medvedeva 2006) based on the intelligent tutoring system design (Anderson et al. 1995) that is used in a variety of other educational settings (Gertner and VanLehn 2000; Koedinger et al. 1997). The system provides immediate feedback via error correction and guidance on immediate steps, thus enabling users to ‘learn by doing’ (Crowley and Gryzbicki 2006). We have previously shown that use of the system is associated with a marked improvement in diagnostic performance (Crowley et al. 2007; El Saadawi et al. 2008), an increase in calibration of confidence to performance (El Saadawi et al. 2008), and a decrease in many types of specific reasoning errors (Payne et al. 2009). A further goal of our research is to use this system to detect and remediate more systematic reasoning errors such as those described as heuristic biases. In particular, we wish to understand when and for whom use of cognitive shortcuts is associated with systematic errors, and to distinguish these cases from those in which heuristic use may be a highly adaptive strategy, so that we intervene selectively during simulation training.

As a first step towards this goal, we sought to (1) define each heuristic and/or bias as a measurable behavior, (2) automate the detection of these behaviors in a computer-based system that provided no immediate feedback, (3) determine the frequency of heuristics and/or biases across multiple levels of training, and (4) determine the degree to which heuristics were used appropriately or inappropriately.

## Methods

### Participants

The study was conducted at two professional medical meetings: (1) the 2009 American Society for Clinical Pathology (ASCP) Annual Meeting and (2) the 2010 United States and Canadian Academy of Pathology (USCAP) Annual Meeting. Recruitment materials (letters and flyers) were emailed to pathology residents, fellows, and 1st and 2nd year attending pathologists through listservs and marketing email lists. All subjects were volunteers and received $200 for the 4 h study. The study was approved by the University of Pittsburgh Institutional Review Board (IRB Protocol # PRO09030260).

### Study design and timeline

During the session, participants used the system as they worked through two domain case sets in (A) subepidermal vesicular dermatitides (SVD) and (B) nodular and diffuse dermatitides (NDD). Each of the case sets contained a total of 20 cases. To control for order effects of the case sets, participants were randomly assigned to start with SVD or NDD case sets. The entire session consisted of five activities: (1) system and study training, (2) study of the first domain, (3) working period for the first domain, (4) study of the second domain, and (5) working period for the second domain. We controlled for time-on-task during each of the five activities. A study period for each sub-domain was included to reduce the risk that biases would be committed due to lack of specialized knowledge. For example, participants may not know that additional diagnoses were in the same differential as a diagnosis associated by them to a set of diagnostic findings. Hence, inclusion of the study period was used us to reduce the possibility of generating over-inflated counts of biases that masked inadequate sub-domain knowledge.

**Fig. 1:**
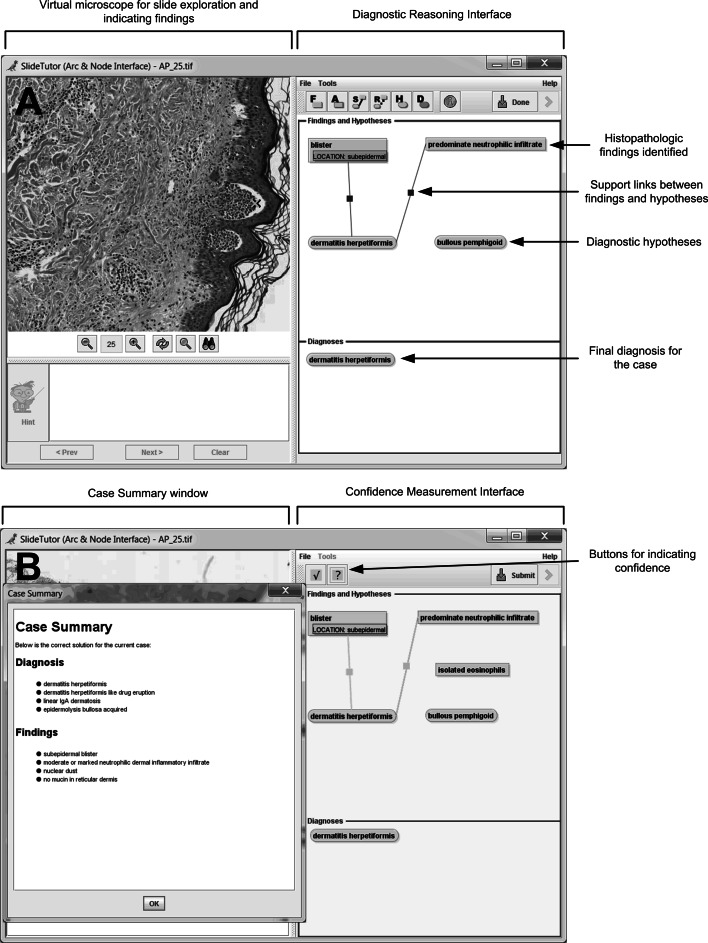
Data collection system showing **a** virtual slide and diagnostic reasoning interface and **b** confidence measurement interface and case summary

We first trained participants to use the data collection system. They watched a 20 min video demonstration of the system, and then practiced using it while being monitored by the trainer. Participants were asked to fully articulate their reasoning using the diagrammatic palette. When they sought for and found specific visual findings during the course of reasoning, they were instructed to identify them using the *Diagnostic Reasoning Interface* (Fig. 1a). Similarly, when they generated a hypothesis or arrived at a diagnosis, they were instructed to specify them. Alternatively, if they did not use such intermediate steps, they were asked not to enter them. The only action that was required by the system was the identification of at least one hypothesis and diagnosis.

Prior to the start of each of the two working periods, participants were asked to review relevant domain study material to limit the possibility that errors committed would be more suggestive of inadequate knowledge rather than heuristic biases. The study material included definitions and images of important findings to look for, and a diagnostic tree showing how findings related to all possible diagnoses. Study periods lasted a total of 20 min each.

Participants were then asked to complete cases at their own pace, during each of the two working periods. Each working period lasted a total of 75 min, and included a maximum of twenty cases. It was stressed to participants that they did not have to complete all of the cases during the working period.

After working through both sets of cases, participants completed a brief survey about their level of training in pathology, including current status (resident, fellow, or clinical practice), years and months completed in that status, and whether they ever had a rotation in dermatopathology or medical training in another country.

### Case selection and sequence

Cases were obtained from the University of Pittsburgh Medical Center (UPMC) slide archive, and from private slide collections. Diagnosis was confirmed by a collaborating dermatopathologist (DJ) prior to use in the study. A total of 20 cases each of SVD and NDD were used. All of the cases used were typical presentations of rare entities. For each case, a knowledge engineer (MC) and an expert dermatopathologist (DJ) collaborated in defining all present findings and locations (case annotation) and in developing the relationships among findings and diagnoses (knowledge base development). The diagnosis included a set of one or more diseases that matched the histopathologic pattern. The system accepted any of the members of this set as the correct diagnosis.

### Data collection

Data on heuristics and biases was collected using a computer-based system, modified from SlideTutor, an intelligent tutoring system used to teach diagnostic surgical pathology (Crowley and Medvedeva 2006). Participants using the system were provided with a virtual microscopic slide to pan and zoom, as well as a *Diagnostic Reasoning Interface* (Fig. 1a) that they could use to diagram their reasoning, including any of the following: (1) pointing to and naming histopathologic findings in the virtual slides, thus creating icons for each finding, (2) selecting diagnostic hypotheses, thus creating icons for each hypothesis, (3) drawing supporting and refuting relationships between findings and hypotheses, with solid linking lines representing supporting relationships and dotted linking lines representing refuting relationships, and (4) recording the final diagnosis as either a single disease or a set of diseases (differential diagnoses). Findings, hypotheses, and diagnoses were selected from a menu of all histopathologic findings and diagnoses applicable to the entire subdomain.

The computer-based system used in this study differed in three ways from the standard SlideTutor system. First, participants *were not provided with feedback* *on individual steps* while working on the cases. Instead, our software ran through all of the study data after collection to determine if each intermediate action was correct or incorrect, and determine when heuristics and/or biases were employed (see Heuristic Definitions and Detection). Second, to measure participant confidence in each action, we used a *Confidence Measurement Interface* (Fig. 1b) that we have previously employed to measure metacognitive gains (El Saadawi et al. 2010). After concluding each case, participants entered the *Confidence Measurement Interface* and were asked to indicate how confident they were about each item they identified by marking findings, hypotheses, diagnoses and relationships in green if they felt sure about them or in yellow if they felt unsure about them. Third, we presented a *Case Summary* window (Fig. 1b) after participants indicated their confidence, which listed the correct diagnosis(es) for the case, and the findings that should have been identified. The case summary was needed because some heuristics (such as *availability* and *gambler’s fallacy*) can only occur if the participant is aware of the diagnosis and findings of previous cases.

All user-system communications were captured from the system in real time using the Foundation for Intelligent Physical Agent (FIPA) agent message standard (Medvedeva et al. 2005). System messages included three basic types of events: (1) low-level human–computer interaction such as pressing a button or selecting a menu item (called InterfaceEvents), (2) combinations of InterfaceEvents that represent the most atomic discrete user action, such as identifying a finding or creating a hypothesis (called ClientEvents), and (3) evaluations of the tutoring system indicating the response of the system to the last participant action (called TutorEvents). TutorEvents included the type of error for incorrect actions and the best-next-step at this point in the problem space. All events were time stamped. To measure heuristic use, we extended the Jess reasoning engine with eight production rules to capture each of the eight heuristics studied. Jess is a rule engine and scripting environment written in Java (“JessRules, available at: http://www.jessrules.com/,” 2012). All data was stored in an Oracle database for analysis.

### Heuristic definitions and detection

We studied eight biases believed to occur in medical decision making: *anchoring* (Croskerry 2002; Ellis et al. 1990; Richards and Wierzbicki 1990), *availability* (Croskerry 2002; Dawson and Arkes 1987; Poses and Anthony 1991), *confirmation bias* (Croskerry 2002; Pines 2006), *gambler’s fallacy* (Croskerry 2002; Tversky and Kahneman 1974), *representativeness* (Croskerry 2002; Payne and Crowley 2008; Tversky and Kahneman 1974), *search satisficing* (Croskerry 2002; Redelmeier and Shafir 1995), *overconfidence* (Berner and Graber 2008; Croskerry and Norman 2008; Friedman et al. 2005) and *underconfidence* (Mann 1993). Definitions of these biases appearing in the literature are often qualitative and therefore do not provide the level of detail required to automate detection. Thus, for each of the biases, we began with accepted qualitative descriptions from the literature and then developed quantitative, algorithmic definitions (Table 1). For two of these biases (*anchoring* and *representativeness*), existing definitions incorporate the notion of appropriate use (i.e. heuristics). Therefore, we also sought to distinguish appropriate use and inappropriate use of the heuristic (bias). Each definition was implemented as a Jess production rule and applied to the entire collected dataset.

**Table 1:** Summary description of heuristics and biases

Heuristic	Definition	Unit of analysis	Heuristic and/or bias	Detection algorithm
Anchoring	Locking on to salient evidence early in the diagnostic process leading to an initial diagnosis	Single case	Heuristic	Participant adds a hypothesis, then adds findings, and then adds a diagnosis supported by the new findings. The diagnosis may be (1) the same as the hypothesis (*sufficient nonadjustment*) or (2) different than the hypothesis (*sufficient adjustment*) as long as the findings support the subsequent diagnosis
			Bias	Participant adds a hypothesis, then adds findings, and then adds a diagnosis that is not supported by the new findings. Participant does not subsequently add hypothesis or diagnosis consistent with all new findings (*insufficient adjustment*)
Availability	The disposition to judge things as either more likely or as frequently occurring if they come to mind readily	Case sequence	Bias	In a sequence of three cases where the third case has a different diagnosis than the first two cases, the participant makes an incorrect diagnosis in the third case. The incorrect diagnosis is identical to the correct diagnosis in the two immediately preceding cases
Confirmation bias	The tendency to look for confirming evidence to support a diagnosis rather than looking for disconfirming evidence to refute it	Single case	Bias	Participants adds an incorrect diagnosis, and then adds findings that support this incorrect diagnosis
Gambler’s Fallacy	The belief that when deviations from expected results occur in repeated independent events, it increases the likelihood of deviations in the opposing direction, leading the clinician to reject a diagnosis because the entity has been observed more frequently than expected in recent cases	Case sequence	Bias	In a sequence of three cases where all three cases have the same diagnosis, the participant makes a diagnosis in the first case, the same diagnosis in the second case, but a different and incorrect diagnosis in the third case. In the first two cases, the participant’s diagnosis may be either correct or incorrect
Representativeness (Type 2)	The tendency to judge an entity against a mental model based on similarity to a prototype, leading the clinican to rigidly associate a feature with a single disease, based on a learned model	Case sequence	Heuristic	Participant adds a finding and then immediately (in the next action) adds a correct diagnosis. This sequence of actions must have been observed more than once during the session. The relationship between the finding and the diagnosis must have been included in the study period
			Bias	Participant adds a finding and then immediately (in the next action) adds an incorrect diagnosis. This sequence of actions must have been observed more than once during the session. The relationship between the finding and the diagnosis must have been included in the study period
Search Satisficing	The tendency to call off a search once something has been found, leading to premature diagnostic closure	Case sequence	Bias	Participant adds a hypothesis and then adds a diagnosis without intervening findings. The diagnosis must be incorrect or the differential diagnosis must be incomplete. The case must be immediately closed. If any findings are added during the case, they must precede the addition of the hypotheses
Overconfidence and underconfidence	The belief in one’s own performance, with extremes representing opposite ends of a spectrum of feeling-of-knowing	Case sequence	Bias	Based on the comparison of participant self assessment to performance for all items. Bias is computed as result of the subtraction of total correct from total sure, divided by total items, and range from +1 (completely overconfident) to −1 (completely underconfident), with an optimum value of zero indicating perfect matching between their confidence and performance

For *anchoring*, *confirmation bias*, *representativeness*, *search satisficing*, *overconfidence,* and *underconfidence,* detection was at the level of a single case. Every individual case in the set represented an opportunity for the heuristic or bias to occur. For these heuristics, a case was considered positive for that heuristic or bias regardless of the number of times the heuristic or bias occured in that case. For *availability* and *gambler’s fallacy*, detection was at the level of a case sequence. We created specific sub-sequences that were designed to provide opportunities for each of these two biases to occur.

Each case could be classified as an instance of more than one heuristic or bias. Definitions for each heuristic follow. A summary of the definitions and sequence of actions required to detect each heuristic is provided in Table 1.

*Anchoring* is the tendency to cognitively lock on to salient features early in the diagnostic process leading to an initial diagnosis (Croskerry 2002; Tversky and Kahneman 1974). *Anchoring* may bias clinicians when they fail to adjust this initial impression in light of later information (Croskerry 2002; Ellis et al. 1990; Richards and Wierzbicki 1990). Our system determined that participants anchored if they entered one or more hypotheses before entering one or more findings. If the added finding supported the reported diagnostic hypothesis or if the participant, after reporting the finding, added one or more diagnoses supported by the findings, our system determined that they correctly used the anchoring heuristic. If, however, the participant did not adjust their initial hypothesis in light of having reported evidence (i.e., findings) that did not support it, we determined that the participant was subject to the anchoring bias. *Anchoring* was evaluated for all cases completed.

*Availability* is the disposition to judge things as either more likely or as frequently occurring if they come to mind readily (Croskerry 2002; Tversky and Kahneman 1974). *Availability* can bias clinicians towards an incorrect diagnosis in an unknown case when it is seen in close proximity to one or more known examples of cases with similar symptoms but unrelated diagnosis (Croskerry 2002; Dawson and Arkes 1987; Poses and Anthony 1991). Our system determined that *availability* bias occurred if the participant incorrectly diagnosed a case as a specific diagnosis or differential diagnoses after being exposed to the case and summary of two immediately preceding cases of that diagnosis. *Availability* bias was evaluated in 6 cases where it could occur based on our case sequence.

*Confirmation bias* is the tendency to look for confirming evidence to support a diagnosis rather than looking for disconfirming evidence to refute it (Croskerry 2002; Nickerson 1998). This bias can lead clinicians to over-value evidence that confirms an incorrect diagnosis (Croskerry 2002; Pines 2006). Our system determined that *confirmation bias* occurred in cases where participants made an incorrect diagnosis, and then immediately identified a finding that supports this incorrect diagnosis. *Confirmation bias* was evaluated for all cases completed.

*Gambler’s fallacy* is the belief that if deviations from expected results occur in repeated independent events, it increases the likelihood of deviations in the opposing direction (Tversky and Kahneman 1974). This is most classically expressed as the expectation that a fair coin toss is more likely to result in a ‘heads’ after a series of repeated ‘tails’. In clinical reasoning, gambler’s fallacy could lead the diagnostician to erroneously reject a diagnosis because the disease has been observed more frequently than expected in recent cases (Croskerry 2002). Our system determined that the *gambler’s fallacy* bias occurred if the participant erroneously diagnosed a case as being different from the two previous cases which depicted the same pattern and diagnosis. *Gambler’s fallacy* bias was evaluated in 10 cases where it could be employed based on our case sequence.

*Representativeness* describes a set of heuristics related to tendencies to judge an entity against a mental model (Tversky and Kahneman 1974). The most commonly recognized form of *representativeness* is the matching of data to a mental model based on the similarity to a prototype, which has been described by Tversky and Kahneman as *Type 2 Representativeness* (Kahnamen and Tversky 1972; Tversky and Kahnamen 1980). In clinical reasoning, *Type 2 Representativeness* may “drive the diagnostician toward looking for prototypical manifestations of a disease, (resulting in) atypical variants being missed” (Croskerry 2003). One indicator of this form of *representativeness* is the degree to which clinicians rigidly associate a finding with a single disease based on a learned model. Because we used cases from a sub-domain not commonly found in the participants’ practice, we hypothesized that one way to test whether this heuristic/bias occurred was by monitoring instances of a “learned model” representing relationships between finding and diagnosis. These relationships were learned by participants during the study periods. Hence, our system determined that *representativeness* occurred when, during the working periods, participants identified the learned finding-diagnosis pair. Moreover, this sequential use of the “learned model” had to be repeated twice before our system attributed the pairing to *representativeness*. Cases were sub-classified as bias when the diagnosis was incorrect or heuristic when the diagnosis was correct. *Representativeness* was evaluated for all cases completed.

*Search satisficing* reflects the tendency to call off a search once something has been found (Simon 1956). In clinical reasoning, this heuristic can lead to premature diagnostic closure (Croskerry 2002; Redelmeier and Shafir 1995). Our system determined that *search satisficing* bias occurred when the following sequence was detected: finding identification (this step was optional), creation of a hypothesis, and acceptance of this hypothesis as the final diagnosis when the diagnosis was either incorrect for the case, or correct but incomplete (other equally plausible diagnoses could be made and hence should have been included in the differential). *Search satisficing* bias was evaluated for all cases completed.

*Overconfidence* and *underconfidence* measure opposite ends of a spectrum of feeling-of-knowing (Nelson 1984). *Overconfidence* is the belief that one’s performance is better than it is, while *underconfidence* is the belief that one’s performance is worse than it is. Recent authors have suggested that *overconfidence* may be an important factor in a variety of diagnostic and treatment errors (Berner and Graber 2008; Croskerry and Norman 2008). To determine confidence, self-assessment data was collected at the end of each case, and was compared to actual performance. Metrics were based on a two-by-two contingency table created for each case by comparing feeling-of-knowing (reported by participants on each finding and diagnosis as ‘sure’ or ‘unsure’) to performance (determined by the system on each finding and diagnosis as ‘correct’ or ‘incorrect’). Thus, findings and diagnoses were weighted equally. For each case, for each participant, we computed the bias as the result of the subtraction of total correct from total sure, divided by total items (Kelemen et al. 2000). Bias scores range from +1 (completely overconfident) to -1 (completely underconfident), with an optimum value of zero indicating perfect matching between their confidence and performance. *Overconfidence* and *underconfidence* biases were evaluated for all cases completed.

**Fig. 2:**
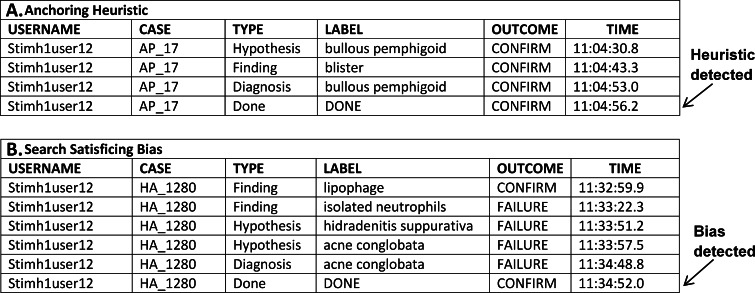
Sample user data showing examples of several heuristics

Figure 2 illustrates sample participant data that classified as heuristic or bias, and the point at which the heuristic or bias was detected by the system. The top portion of the figure shows an example of data coded as the *anchoring heuristic.* The participant identified ‘bullous pemphigoid’ as a hypothesis, added the finding ‘blister’, and made ‘bullous pemphigoid’ their final diagnosis. The participant’s diagnosis of ‘bullous pemphigoid’ is the same disease they were already considering, so no adjustment occurred. Because ‘blister’ supports ‘bullous pemphigoid’, it was appropriate for the user to not adjust. The bottom portion of Fig. 2 illustrates an example of the *search satisficing* bias. The user identified several findings, added ‘acne conglobata’ as a hypothesis, indicated ‘acne conglobata’ as the final diagnosis (which is incorrect) and closed the case.

### Data analysis

Task measures, including number of cases completed, and percent correct of diagnoses and findings, were analyzed by level of training and by sub-domain. Analysis by level of training was carried out by grouping both sub-domains (NDD and SVD). On the other hand, analysis by sub-domain was carried out by grouping participants (and hence no distinctions among levels of training were taken into account). Descriptive statistics included mean and standard deviation. One-way ANOVA was used to compare differences by level of training. A Shapiro–Wilk test was used to test for normality. For non-normal measures, descriptive statistics included median and standard deviation from the median. Kruskal–Wallis test was used to compare differences by level of training. Mann–Whitney *U* test was used to compare differences by sub-domain in the case-based analyses, and to compare the frequency of heuristic and bias use. Significance was set at the 0.05 level. All analyses were performed in IBM SPSS Statistics v19.

## Results

### Participants

Participants included a total of 71 Pathology residents, fellows, and 1st and 2nd year attending pathologists from institutions across the United States. Subjects were divided into three levels of training: 22 1st and 2nd year residents (Level 1), 26 3rd and 4th year residents (Level 2), and 23 fellows and 1st and 2nd year attending pathologists (Level 3). Of the 71 total participants, 34 had previously participated in a Dermatopathology rotation.

### Task metrics

Across all 71 participants, the study produced a total of 2230 cases for analysis. Participants completed an average of 15.7 subepidermal vesicular dermatitis cases, and 15.7 nodular and diffuse dermatitis cases (Table 2). One-way ANOVA results indicated a significant difference in the number of nodular and diffuse dermatitis cases seen by level of training, *F*(2, 68) = 4.05, *MSE* = 20.70, *p* = .02, η_p_^2^ = .11. Post hoc analyses using the Scheffé test indicate this difference arises due to Level 1 participants seeing significantly fewer cases than Level 3 (*p* = .04), and marginally fewer cases than Level 2 (*p* = .07). However, no differences existed in the number of subepiermal vesicular dermatitis cases seen by level of training, *F*(2, 68) = 1.80, *MSE* = 18.75, *p* = .17, η_p_^2^ = .05.

**Table 2:** Task metrics (overall and by level of training)

	All (N = 71)		Level of training					
			Level 1 Y1 and Y2 residents (N = 22)		Level 2 Y3 and Y4 residents (N = 26)		Level 3 Fellows, Y1 and Y2 attendings (N = 23)	
	Mean	SD	Mean	SD	Mean	SD	Mean	SD
Total number of cases completed	31.4	6.8	27.8	6.3	32.5	7.1	33.7	5.7
Subepidermal vesicular (only)	15.7	4.4	14.4	4.5	15.9	4.2	16.8	4.4
Nodular and diffuse (only)	15.7	4.7	13.4	5.0	16.5	4.5	16.9	4.2

### Analyses by participant: accuracy of findings and diagnoses

Mean diagnostic accuracy achieved was 36.0 % for subepidermal vesicular dermatitides (SVD), and 39.1 % for nodular and diffuse dermatitides (NDD) (Table 3). Mean accuracy of findings was 64.1 % for SVD, and 68.0 % for NDD (Table 3). Overall accuracy is similar to previously published pre-intervention results from our laboratory (Crowley et al. 2007; El Saadawi et al. 2008).

**Table 3:** Diagnostic and finding accuracy (overall and by level of training)

	All (N = 71)		Level of training						ANOVA	
			Level 1 Y1 and Y2 residents (N = 22)		Level 2 Y3 and Y4 residents (N = 26)		Level 3 Fellows, Y1 and Y2 attendings (N = 23)		*F*	*p*
	Mean (%)	SD (%)	Mean (%)	SD (%)	Mean (%)	SD (%)	Mean (%)	SD (%)		
Overall diagnostic accuracy	37.0	10.4	38.6	9.9	34.2	10.0	38.7	10.9	1.56	.22
Subepidermal vesicular (only)	36.0	14.3	36.8	13.0	35.6	14.0	35.8	16.4	0.04	.96
Nodular and diffuse (only)	39.1	12.6	41.9	10.3	33.2	12.4	43.0	12.9	4.92	.01
Overall finding accuracy	65.9	7.9	66.3	8.4	64.1	8.9	67.5	5.9	1.18	.31
Subepidermal vesicular (only)	64.1	9.3	62.7	10.2	63.9	9.9	65.8	7.6	0.62	.54
Nodular and diffuse (only)	68.0	11.7	70.6	10.5	64.4	14.3	69.5	8.8	1.90	.16

One-way ANOVA results indicated a significant difference in diagnostic accuracy for nodular and diffuse dermatitis cases by level of training. *F*(2, 68) = 4.92, *MSE* = 143.41, *p* = .01, η_p_^2^ = .13. Post hoc analyses using the Scheffé test reveals that Level 2 participants had significantly lower diagnostic accuracy than Level 3 participants (*p* = .02), and marginally lower accuracy than Level 1 participants (*p* = .05). There were no significant differences in diagnostic accuracy for subepidermal vesicular dermatitides.

### Analyses by participant: bias and heuristic frequency

Across all cases analyzed, the frequency of the heuristics (computed as total occurrences/opportunities) was highest for *search satisficing* followed by *availability*, *anchoring*, *gambler’s fallacy*, and *representativeness* (Tables 4 and 5). *Confirmation bias* was observed least frequently (Table 4).

**Table 4:** Frequency of occurence of Availability, Confirmation, Gambler’s Fallacy and Satisficing

	All		Level of training					
			Level 1 Y1 and Y2 residents		Level 2 Y3 and Y4 residents		Level 3 Fellows, Y1 and Y2 attendings	
	Median	SD	Median	SD	Median	SD	Median	SD
Availability bias								
Number of cases	1.0	1.00	1.0	0.85	1.0	1.26	1.0	0.83
% of possible cases	20.00	21.01	20.00	22.02	20.00	22.77	20.00	18.79
Confirmation bias								
Number of cases	0.0	0.43	0.0	0.22	0.0	0.35	0.0	0.64
% of possible cases	0.0	1.34	0.0	0.87	0.0	1.31	0.0	1.75
Gambler’s Fallacy bias								
Number of cases	0.0	0.94	0.0	0.79	1.0	0.85	1.0	0.80
% of possible cases	0.0	11.39	0.0	11.38	10.00	9.22	10.00	8.21
Satisficing bias								
Number of cases	6.0	8.04	3.50	5.09	9.50	9.63	10.0	6.32
% of possible cases	22.50	20.82	13.81	16.55	26.67	24.51	30.00	18.68

Cases classified as bias per participant (overall and by level of training)

Legend: For each user, the denominator used for percent of possible cases is the number of cases that the user completed in which the heuristic could occur. Therefore, the denominator differs across users

**Table 5:** Frequency of occurence of Anchoring and Representativeness

	All		Level of training					
			Level 1 Y1 and Y2 residents		Level 2 Y3 and Y4 residents		Level 3 Fellows, Y1 and Y2 attendings	
	Median	SD	Median	SD	Median	SD	Median	SD
Anchoring heuristic								
Number of cases	2.0	2.29	2.0	2.72	2.0	2.02	2.0	2.23
% of possible cases	5.26	9.64	5.71	12.15	5.06	9.35	5.13	7.27
Anchoring bias								
Number of cases	2.0	3.25	2.50	3.46	1.0	3.87	3.0	2.50
% of possible cases	5.88	12.24	8.84	15.19	4.00	13.71	7.50	7.51
Representativeness heuristic								
Number of cases	1.0	1.87	1.0	2.05	1.0	1.62	1.0	2.04
% of possible cases	3.45	6.32	4.65	7.08	3.33	5.12	3.45	6.75
Representativeness bias								
Number of cases	0.0	0.94	0.0	1.07	0.0	1.00	0.0	0.77
% of possible cases	0.0	3.29	0.00	3.85	0.00	3.48	0.00	2.57

Cases classified as heuristic versus bias per participant (overall and by level of training)

Legend: For each user, the denominator used for percent of possible cases is the number of cases that user completed in which the heuristic could occur. Therefore, the denominator differs across users

We did not expect heuristic errors to be normally distributed. Therefore, we conducted Shapiro–Wilk tests for each bias and heuristic studied (Shapiro–Wilk, *W* = 0.44–0.88, *p* < .001 in all cases). Table 4 provides median and standard deviation from the median for *availability*, *confirmation bias*, *gambler’s fallacy*, and *search satisficing*, overall and by level of training. Kruskal–Wallis results indicate that only *search satisficing* showed an effect for level of training, χ^2^(2, N = 71) = 7.50, *p* = .02, η^2^ = .11. Mann–Whitney *U* tests indicate this difference is due to Level 1 participants satisficing less frequently than both Level 2 participants, *z* = −2.65, *p* = .01, *r* = .38; and Level 3 participants, *z* = −2.04, *p* = .04, *r* = .30. The number of cases in which biases occurred did not differ by level of training for *availability*, χ^2^(2, N = 71) = 0.83, *p* = .66, η^2^ = .01; *confirmation bias*, χ^2^(2, N = 71) = 4.61, *p* = .10, η^2^ = .07; or *gambler’s fallacy*, χ^2^(2, N = 71) = 2.36, *p* = .31, η^2^ = .03.

Table 5 provides similar descriptive statistics for *representativeness* and *anchoring*, overall and by level of training. In these cases, it was possible to compute frequencies of the behavior associated with both appropriate use of the heuristic as well as bias. Mann–Whitney *U* tests show that there was not a significant difference between the frequency of heuristic and bias use for *anchoring*, *z* = −0.29, *p* = .77, *r* = .02. In contrast, heuristic use was significantly more frequent than bias use for *representativeness*, *z* = −4.07, *p* < .001, *r* = .34. There were no significant differences in the use of the *anchoring* heuristic, χ^2^(2, N = 71) = 0.32, *p* = .85, η^2^ = .004; or bias, χ^2^(2, N = 71) = 1.13, *p* = .57, η^2^ = .02, by level of training. Additionally, no differences were observed in the use of *representativeness* heuristic, χ^2^ (2, N = 71) = 0.10, *p* = .95, η^2^ = .001; or bias, χ^2^ (2, N = 71) = 0.10, *p* = .95, η^2^ = .001, by level of training.

Given our definitions and the case sequence, 51.2 % of cases in which a diagnostic error occurred were associated with one or more of the measured biases (Table 6). In contrast, 21.0 % of cases in which no diagnostic error occurred were associated with one or more of the measured heuristics.

**Table 6:** Heuristic and bias use by cases with correct and incorrect diagnoses

	All	Level of training		
		Level 1 Y1 and Y2 residents	Level 2 Y3 and Y4 residents	Level 3 Fellows, Y1 and Y2 attendings
Percentage of cases with a final *incorrect* diagnosis (mean count)	67.6 % (21.2)	68.4 % (19.0)	69.7 % (22.6)	64.6 % (21.8)
Percentage of cases with a final *incorrect* diagnosis in which a bias occurred (mean count)	51.2 % (10.9)	43.5 % (8.3)	57.1 % (12.9)	50.5 % (11.0)
Percentage of cases with a final *correct* diagnosis (mean count)	32.4 % (10.2)	31.6 % (8.8)	30.3 % (9.9)	35.3 % (11.9)
Percentage of cases with a final *correct* diagnosis in which a heuristic occurred (mean count)	21.0 % (2.1)	24.4 % (2.1)	19.5 % (1.9)	20.1 % (2.4)
Frequency of bias occurrence when the diagnosis was incorrect (normalized for opportunity)				
Percentage of cases with diagnostic error where *Anchoring* was detected (mean count)	10.7 % (2.3)	12.7 % (2.4)	10.4 % (2.4)	9.4 % (2.0)
Percentage of cases with diagnostic error where *Availability* was detected (mean count)	32.7 % (1.0)	30.8 % (0.8)	34.7 % (1.2)	31.6 % (1.0)
Percentage of cases with diagnostic error where *Confirmation Bias* was detected (mean count)	0.7 % (0.2)	0.3 % (0.1)	0.5 % (0.1)	1.4 % (0.3)
Percentage of cases with diagnostic error where *Gambler’s Fallacy* was detected (mean count)	14.0 % (0.6)	11.0 % (0.4)	16.6 % (0.7)	13.8 % (0.7)
Percentage of cases with diagnostic error where *Representativeness* was detected (mean count)	2.4 % (0.5)	3.1 % (0.6)	2.3 % (0.5)	1.9 % (0.4)
Percentage of cases with diagnostic error where *Search Satisficing* was detected (mean count)	33.5 % (7.1)	23.2 % (4.4)	41.0 % (9.3)	33.3 % (7.3)
Frequency of heuristic occurrence when the diagnosis was correct (normalized for opportunity)				
Percentage of correctly diagnosed cases where *Availability* heuristic was detected (mean count)	9.9 % (1.0)	13.5 % (1.2)	7.8 % (0.8)	9.5 % (1.1)
Percentage of correctly diagnosed cases where *Representativeness* heuristic was detected (mean count)	12.1 % (1.1)	11.6 % (1.0)	12.7 % (1.2)	11.8 % (1.3)

**Fig. 3:**
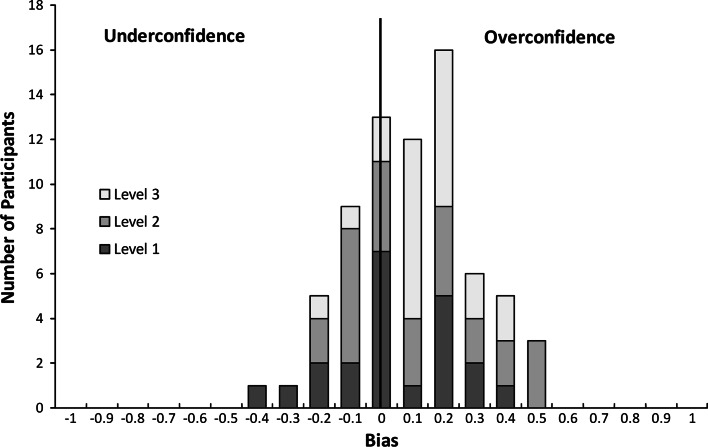
Distribution of bias scores. Number of participants (by level of training) with average bias scores at 0.1 intervals. Bias scores on *x* axis reflect center of interval. The optimum point at zero is marked with a vertical line

We also examined the distribution of *overconfidence* and *underconfidence* biases. Figure 3 shows the distribution of bias scores by level of training. Confidence scores were normally distributed, with a range of −0.41 to +0.49, mean = 0.10 and standard deviation = 0.19. Results indicated that participants were overconfident, *t*(70) = 4.39, *p* < .001, *d* = 0.52.

### Analysis by sub-domain: accuracy of diagnoses, bias and heuristic frequencies

For analysis by sub-domain, we used cases as the unit rather than participants. Participants made slightly more diagnostic errors in the cases in the SVD sub-domain (median 70.3 %) than in the NDD domain (median 62.0 %), but this difference was not statistically significant by Mann–Whitney *U* test, *z* = −0.41, *p* = .69, *r* = .06. Comparing heuristic and bias usage between sub-domains, significant differences exist only in the use of *search satisficing*, which occurred more frequently in the NDD cases (median 38.1 %) than in the SVD cases (median 19.0 %), *z* = −4.14, *p* < 0.001, *r* = .65.

*Availability* and *gambler’s fallacy* could only occur in select cases in each sub-domain. Therefore, no statistical analysis could be conducted for these heuristics. However, there was a trend for participants to commit *availability* more frequently in the SVD sub-domain (median 34.3 %) than in the NDD sub-domain (median 17.9 %). The reverse trend was observed for *gambler’s fallacy* (median NDD = 7.15 %; median SVD = 6.10 %).

## Discussion

In this study, we ‘turned off’ the educational intervention of an existing intelligent tutoring system to collect data on heuristics and biases as pathologists examine a set of virtual slides. An important first goal of this research was to develop computable definitions for a set of well-known heuristics, and then implement these definitions within the hypothetico-deductive framework used in our tutoring system. The resulting definitions are general enough to be used by others who may be developing computer-based approaches to remediating cognitive errors.

We observed that diagnostic errors in this area of dermatopathology are frequent, and they are not obviously correlated with level of training. Results are quite similar to previous pre-test performance results that we have published for similar tasks (Crowley et al. 2007; El Saadawi et al. 2008). Dermatidides are a difficult diagnostic area, often seen only by subspecialist dermatopathologists or dermatologists in academic medical centers. One or more of these heuristic biases occurred in approximately half of the diagnostic errors detected in this study, suggesting they are a potentially important source of error for participants using our training systems.

Our results show that *search satisficing* and *availability* were the biases most frequently observed among participants, with other biases occurring less often. Search satisficing has been extensively explored in Radiology. Berbaum et al. (1990, 2000) have repeatedly shown that under certain conditions, detection of one abnormality significantly interferes with detection of another abnormality on the same case. Although we have not examined any relationship effects in this current study, Berbaum’s research suggests that detection of a secondary abnormality is inversely proportional to the severity of the first lesion detected (Berbaum et al. 2001).

Here we observed significant differences by level of training only for the *search satisficing* bias, where participants with more training were more likely to satisfice. These findings contradict those by Heller et al. (1992), which included 1st-, 2nd- and 3rd-year residents and found that 1st-year residents were more likely to satisfice than their more experienced colleagues. One possible reason for this discrepancy is that Heller et al. asked residents to arrive at a diagnosis of cases presented as clinical vignettes which described ficticious encounters with patients, whereas our participants were presented with actual digitized clinical material. Thus, it can be argued that the information content was significantly greater in our study than the one conducted by Heller et al.

In our study, other heuristics and biases appeared to be independent of level of training. This result differs from those reported by Mamede et al. (2010), which studied the diagnostic reasoning processes of 1st and 2nd-year internal medicine residents. These authors found that 2nd-year residents committed the availability bias significantly more than their 1st year colleagues. It is possible that the discrepancy between our studies stems from the fact that we grouped 1st- and 2nd- year residents in a single category, thus eliminating our ability to detect any differences between them. We also found that *overconfidence* was more prevalent than *underconfidence*, a result that confirms similar findings among clinicians (Berner and Graber 2008; Friedman et al. 2005) and others (Brenner et al. 1996). Analysis of heuristics and biases by case suggested that there were significant differences for commission of *search* *satisficing* between the NDD and SVD sub-domains, with participants ending case interpretation early much more often in the NDD cases.

In cases where we could detect both appropriate use and bias, we found no differences for use of *anchoring*, but significant differences for use of *representativeness*, with the heuristic occurring almost 4 times as often as the bias. A recent study by Payne provides empirical evidence that *anchoring* is in fact associated with diagnostic accuracy among medical students and residents reasoning in the domain of general internal medicine (Payne 2011). Our results add additional support for the viewpoint that heuristics may be more valuable than problematic (Eva and Norman 2005). Further debate about the relative costs and benefits of heuristic application may not be fruitful. A better goal for future research should be to determine what characteristics of individuals and tasks lead to these errors and how we can recognize and diminish the errors without sacrificing the benefits of heuristic use.

This manuscript introduces a new method for investigating heuristic and bias application that can be immediately applied to educational systems. The method supports prospective, laboratory-based studies and automated analysis, and therefore enables the researcher to experimentally manipulate parameters of interest in order to observe their effect on clinical reasoning and diagnostic errors. In this study, the analytic phase of heuristic and bias detection was conducted after the data was collected. However, during normal operation, our intelligent tutoring system collects this data in real-time as the student works, producing a model of heuristic and bias usage that is dynamic and learner-specific. Now that we can automatically detect these errors, we are able to investigate interventions that may modify them. We are currently testing two methods for enhancing metacognitive awareness and reducing overconfidence—considering alternatives and playing back mistakes. In future work, we expect to test other interventions for specific heuristics.

This study was limited in a number of ways. First, our definitions were based on descriptions available in the patient safety and decision-making literature, as well as the psychological literature. In many cases, we needed to establish thresholds for classification. For example, both *gambler’s fallacy* and its counterpart bias *availability* required that we select a number of cases immediately preceding the case of interest. Unfortunately, there were few previous definitions of sufficient detail to guide us. In selecting thresholds, we were guided by the principle of balance, attempting to be neither overly liberal nor overly conservative. However, our results are understandably a product of our definitions, and may not apply to other domains or tasks. We hope that our efforts encourage other researchers to more systematically and quantitatively define heuristics and biases in order to interpret data across studies and domains, and also to advance our ability to automatically detect and remediate these errors.

Second, despite our attempts to make the study task as similar as possible to the authentic task, we used a laboratory-based observational design in a difficult diagnostic area for a single medical specialty. Consequently, our results may not generalize outside the laboratory or across other clinical specialties and tasks. Furthermore, the use of the diagnostic reasoning interface may itself have affected the cognitive processing as participants performed the task. Additionally, the nature of a laboratory-based design may not sufficiently reproduce the time pressure and other performance demands of clinical practice and therefore may underestimate heuristic use. Our results also depended on the ability of participants to articulate their cognitive states. For example, if a participant identified a hypothesis but then neglected to identify a finding that they were actively searching for afterwards, our system could not identify a possible *confirmation bias*. This may have also led us to underestimate the heuristic and bias frequency in some cases.

Third, this study used an expert dermatopathologist’s opinion, in the form of finding and diagnosis annotations on each slide, as the standard against which the participants were measured. While this approach is typical of observer performance studies, we note that a gold standard encompassing opinions of multiple experts could be expected to enhance the validity of the results. With regard to the reliability of the heuristic measurements based on this expert standard, we expect that the automated method we developed should naturally be associated with low variability when compared with human scoring.

Despite the limitations, our results provide important insights for developers of intelligent medical training systems (Crowley and Gryzbicki 2006). Most importantly, we were able to build a system that automatically detects a set of heuristics and biases during reasoning, within the typical graphical user interface of our educational system (Crowley et al. 2007; El Saadawi et al. 2008). Our results indicate that we can detect heuristics and biases frequently even when participants are not receiving corrective immediate feedback. This supports the validity of heuristic detection in the more rigidly enforcing feedback environments used in intelligent tutoring systems. Additionally, our observation that biases differ in frequency provides us with rational priorities for designing such interventions. Furthermore, the frequency with which heuristics are appropriately applied produces significant challenges for educators as well as educational system designers. In many ways, intelligent medical training systems provide the ideal environment in which to attempt such intervention because the system knows the outcome in all cases, and can therefore distinguish between these two scenarios while providing “repeated practice and failure with feedback” (Croskerry and Norman 2008). Finally, the finding that participants were often inaccurate in matching confidence to performance validates ongoing efforts (El Saadawi et al. 2010) to incorporate metacognitive interventions (Berner and Graber 2008) in order to reduce diagnostic error. As it is said, “to err is human” (Institute of Medicine (U.S.) 2000), but harm resulting from error can be greatly reduced when clinicians learn to resist the “fog of misplaced optimism”(Berner and Graber 2008).
